# Catastrophe Theory Applied to Neuropsychological Data: Nonlinear Effects of Depression on Financial Capacity in Amnestic Mild Cognitive Impairment and Dementia

**DOI:** 10.3390/e24081089

**Published:** 2022-08-07

**Authors:** Dimitrios Stamovlasis, Vaitsa Giannouli, Julie Vaiopoulou, Magda Tsolaki

**Affiliations:** 1 School of Philosophy and Education, Aristotle University of Thessaloniki, 54124 Thessaloniki, Greece; 2 1st Department of Neurology, School of Medicine, Faculty of Health Sciences, Aristotle University of Thessaloniki, 54634 Thessaloniki, Greece; 3 Department of Education, University of Nicosia, Nicosia 2417, Cyprus; 4 School of Psychology, Aristotle University of Thessaloniki, 54124 Thessaloniki, Greece; 5 Alzheimer Hellas, 54643 Thessaloniki, Greece; 6 Laboratory of Neurodegenerative Diseases, Center for Interdisciplinary Research and Innovation (CIRI-AUTh), Balkan Center, Buildings A & B, Thessaloniki, Aristotle University of Thessaloniki, 10th km Thessaloniki-Thermi Rd, P.O. Box 8318, 54124 Thessaloniki, Greece

**Keywords:** cusp catastrophe, complexity, nonlinear dynamics, financial capacity, amnestic mild cognitive impairment, depressive symptoms

## Abstract

Financial incapacity is one of the cognitive deficits observed in amnestic mild cognitive impairment and dementia, while the combined interference of depression remains unexplored. The objective of this research is to investigate and propose a nonlinear model that explains empirical data better than ordinary linear ones and elucidates the role of depression. Four hundred eighteen (418) participants with a diagnosis of amnestic MCI with varying levels of depression were examined with the *Geriatric Depression Scale* (GDS-15), the *Functional Rating Scale for Symptoms of Dementia* (FRSSD), and the *Legal Capacity for Property Law Transactions Assessment Scale* (LCPLTAS). Cusp catastrophe analysis was applied to the data, which suggested that the nonlinear model was superior to the linear and logistic alternatives, demonstrating depression contributes to a bifurcation effect. Depressive symptomatology induces nonlinear effects, that is, beyond a threshold value sudden decline in financial capacity is observed. Implications for theory and practice are discussed.

## 1. Introduction

### 1.1. The Psychocognitive Framework

This section focuses on explaining the psychocognitive framework that hosts the present investigation, which has a predominately methodological orientation. Thus, from the existing extensive literature on dementia, amnestic mild cognitive impairment, and the factors involved in defining, measuring, and treating them, only the most relevant pieces will be cited, which will adequately familiarize the reader with the phenomenon under study.

An issue that has become crucial in modern societies, since it relates to legal implications, is the assessment of the financial capacity of older adults with psychocognitive problems [1,2,3]. The matter concerns a number of specialists, including not only clinical neuropsychologists and forensic psychiatrists, but also judges and lawyers, while there is an increasing theoretical interest in proposing models for describing and predicting empirical results. Although there is not a consensus among researchers about defining and measuring financial capacity [4,5,6], a predominant model (Marson’s model) [7] proposes an effective way to deal with the multidimensionality of the latent variable in question, and it is acceptable for the legal systems in most countries. Conceptually, the model includes two components: the first encompasses the financial activity of a general domain of functioning, and the second takes into consideration specific financial abilities tasks. This general model has inspired potential endeavors for developing assessment instruments in different countries, given that the underlying process is culture-specific [8]. The interest in developing such tools aims at their implementation in clinical assessments, as direct measurements of relevant neuropsychological deficits. Analogous endeavors have been realized in other domains by examining different mental resources, such as memory skills or verbal fluency [9,10]. Given the abovementioned legal implications, the central idea continues to inspire a growing concern, specifically for the financial capacity, since it has been proven highly susceptible to Alzheimer’s disease (AD) and related disorders [11]. In this direction, lately, a financial capacity test for the Greek population, namely the *Legal Capacity for Property Law Transactions Assessment Scale* (LCPLTAS), was developed and validated [12], with psychometric properties that allow the performance of both healthy older adults and those suffering from different types and stages of dementia to be investigated. It should be noted that individuals with mild cognitive impairment (MCI) are also distinguished via this test. Relevant diagnostic cognitive tests, such the Mini-Mental State Examination (MMSE), were used to predict financial capacity performance, enhancing the validly of the LCPLTAS. In addition, two more instruments, the *Geriatric Depression Scale* (GDS-15) and the *Functional Rating Scale for Symptoms of Dementia* (FRSSD) were used as predictor variables in order to associate LCPLTAS scores with the other scales.

### 1.2. The Effects of Psychocognitive Resources on Financial Capacity

Research on psychocognitive performance, based on empirical evidence, has established a number of relationships among latent factors related to some mental deficits.

Patients with amnestic mild cognitive impairment (aMCI) are found to be inferior performers in financial capacity tasks compared to healthy individuals [13,14], and the anticipated decline over time in MCI converters is significantly greater than that of the MCI non-converters or healthy control cases [13]. However, there are circumstances where additional factors can concomitantly affect financial capacity, such as comorbid depression. Research has shown that decline in financial capacity in Alzheimer’s Disease, Parkinson’s Disease, and vascular dementia is observed, specifically when depression is identified during neuropsychological assessment [15,16]. Moreover, studies have supported declining and impaired financial capacity in aMCI individuals [13,14], while some empirical evidence for financial capacity in aMCI with concurrent depressive symptomatology (aMCI-D) has been provided [17].

Regarding methodological issues, all relevant research has been promoted via traditional approaches with linear statistical modeling, the limitations of which are already well known [18]. The present endeavor, fostering the meta-theoretical framework of complexity theory and nonlinear dynamics, aimed to test the nonlinear hypothesis in psychocognitive performance by applying catastrophe theory and implementing financial capacity, GDS, and FRSSD. 

Any neuropsychological process is characterized by an inherent complexity. The involved latent constructs, such as financial capacity, are also complex, involving a variety of mental functions, which are operationalized by specific ability tests (e.g., arithmetic, counting coins/currency, paying bills) and judgment decision-making skills [14,15]. All involved mental resources (such as working memory and logical thinking) interact with each other in time via a dynamical process where, in addition to the positively contributing components, counteracting variables and inhibitory factors operate as moderators leading to deteriorated outcomes. Based on the evidence, depression is a moderator factor, which, when combined with additional deficits, leads to a worse performance. It is reasonable to consider that depression not only in AD, but also in other neurocognitive disorders, is a moderator factor for financial capacity [19,20]. This is a hypothesis though that hasn’t received systematic investigation providing a coherent and interpretable model that describes the phenomenon.

To this end, the present article proposes a novel approach in exploring medical data in this area by fostering complexity theory and nonlinear dynamics. It is a fundamental theoretical consideration that the latent constructs involved in neuropsychological processes are dynamically interacting and the emergent behavior is described by the notion of complex adaptive systems (CASs) [21]. In a CAS, the behavior is deemed as inherently nonlinear, and changes can often be discontinuous and unpredictable. Complexity science has already gained considerable attention in social sciences [22,23], behavioral sciences [24,25,26,27,28], and life and medical sciences [29,30,31,32]. The present endeavor employs catastrophe theory for modeling financial capacity as the state variable dependent measure, while the Geriatric Depression Scale (GDS-15) and the Functional Rating Scale for Symptoms of Dementia (FRSSD) are the predictor variables. Elements of catastrophe theory are presented in the following section.

### 1.3. Catastrophe Theory

Catastrophe theory as a mathematical theory was founded on the works of Thom [33] and Arnold [34] and concerns the classification of the equilibrium behavior of dynamical systems in the neighborhood of singularities. It proves that, at these critical points, the system can be locally modeled by seven elementary catastrophes, from which cusp catastrophe is the most known and applicable [35]. Catastrophe theory presupposes a dissipating or potential-minimizing system, and the cusp model is expressed by the first derivative of a potential function, *U*, with respect to the outcome, *y*, by Equation (1):

(1) $$\frac{\partial U\left(y,a,b\right)}{\partial y}=y^{3}-by^{}-ay$$

By setting ∂U(y,a,b)/∂y=0, the resulting equilibrium function is represented by the three-dimensional surface as a function of the two control parameters (α and *b*).

**Figure 1:**
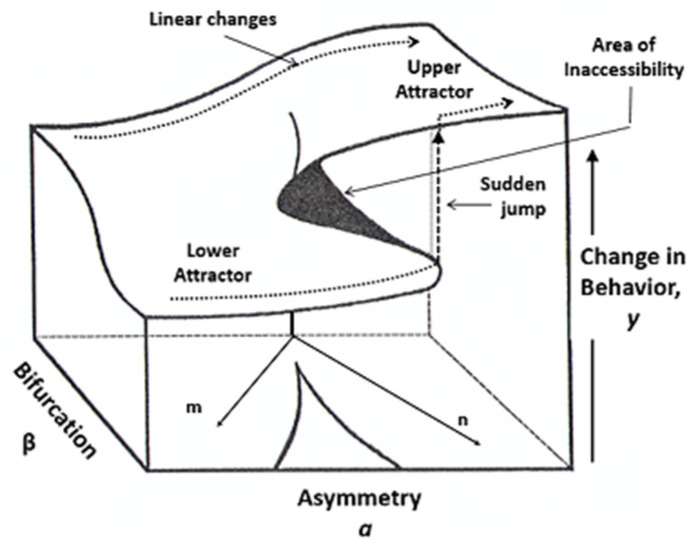
Cusp catastrophe surface.

The development of stochastic catastrophe theory, which is based on the initial work of Cobb [36], allows for testing the relevant models with empirical data. Catastrophe theory is an area of complex dynamical systems and has shown high applicability in behavioral science. The notion of a potential-optimization process is compatible with a neuropsychological system, since it could be considered as pursuing the optimization of some function, e.g., related to adaptation or cognitive dissonance. The description of the cusp model is made via the response surface (Figure 1), where its fundamental features can be observed, such as bimodality, hysteresis, inaccessibility area, divergence, bifurcation, and sudden jumps [37]. The above phenomenology is interpreted via the underlying self-organization processes [38] and is theoretically connected to other areas of nonlinear sciences, such as Prigogine’s non-equilibrium [39,40] and Haken’s synergetics [41,42].

The interpretation of the cusp model is facilitated by the three-dimensional response surface (see Figure 1), which demonstrates the geometry of behavior. At the back region of the surface, where the bifurcation, *b*, has low values, the surface is smooth and a linear relationship between the state variable (dependent measure) and the asymmetry*, a*, holds. In the forward-facing part, the surface folds and the two regions, i.e., the upper and the lower parts, appear, representing the two behavioral modes that in the language of CDS are called *attractors*. At this region, the probability density function of the empirical data becomes bimodal, whereas, in the area between the two modes, the behavior is unlikely to occur, and it is called the inaccessibility area. Thus, in this region, changes can only occur as jumps or transitions between the two behavioral attractors. Mathematically, these changes are called *discontinuities* and the splitting of the system into two states or different modes of behaviors consists of a bifurcation [43]. Looking from the front of the surface, a sigma-like feature, the *hysteresis effect*, can be observed. These are dynamic effects occurring when the bifurcation variable, *b*, goes beyond a critical value. It is pertinent to emphasize that bifurcation characterizes only nonlinear systems and is considered as a fingerprint of complexity [38]. 

## 2. Materials and Methods

### 2.1. Rationale and Research Hypotheses 

In this research and for the relevant diagnoses, well-known neuropsychological assessment tools, such as the GDS, FRSSD, MMSE, and LCPLTAS, were implemented. The instruments operationalize specific psychological resources and are used concomitantly to consolidate conclusions and to help make decisions. The validation of the LCPLTAS [12] was supported with its functional relationships with the rest of the instruments, while the statistical methodology was based on linear modeling. Considering the epistemological and methodological limitations of the traditional linear approaches [18], this research was initiated to examine the applicability of catastrophe theory in this area and to provide insights about the theoretical and practical implications. This neuropsychological endeavor is apparently inductive in nature, since it is a new application in the field, encompassing a supplementary analysis of available data. However, it is also theory-driven, because it is based on the theory of complex adaptive systems (CASs), which is used here to reexamine the outcomes of dynamical processes, such as the neuropsychological processes taking place in assessment procedures. The cognitive factors involved in financial problem solving do not act as parts of a mechanical system, where the outcome can be expressed as a linear function of the contributing mechanisms [18,44,45]. As parts of a CAS system, these components act with no predetermined scenario, but execute their tasks via an iterative dynamical process. The potential nonlinearity can lead to changes encompassing sudden shifts, discontinuities, or transitions, which can be captured by catastrophe theory models. In the present research framework, among the psychological resources involved, *depression* is known as a moderator factor of financial capacity, competing against the positively acting resources. As an inhibitory agent, depression is a potential factor for inducing nonlinear effects. 

To this end, the research hypotheses posited in this endeavor concern the potential role of depression in financial capacity, along with testing the applicability of catastrophe theory in neuropsychology, and are stated as follows:

(1) The effect of the GDS and FRSSD on the LCPLTAS can be described via a cusp catastrophe model.
(2) The GDS is the main candidate for acting as a bifurcation factor.
(3) Both the FRSSD and GDS could contribute to both the asymmetry and the bifurcation factors.

### 2.2. Participants and Measures

The participants were 418 Greek adults (68.2% women), whose age ranged from 45 to 98 years (*mean* = 72.55, *SD* = 8.08, *median* = 72.0). The mean years of education was 8.61 years (*SD* = 4.41, *median* = 6.0). A total of 34.4% were healthy control individuals, while the rest were diagnosed with varying degrees of AD and cognitive impairment. This sample composition ensures large variances in the measured construct and facilitates the variable-centered analyses. The neuropsychological assessments were carried out at the Memory Clinic of Papanikolaou General Hospital and elderly daycare centers during 2012–2016. Written informed consent from each participant was obtained and the study was approved by the Ethics Committee of the Aristotle University of Thessaloniki (protocol code 2.27/3/2013) [12], while the research was performed according to the Declaration of Helsinki. 

Financial capacity was assessed with the *Legal Capacity for Property Law Transactions Assessment Scale* (LCPLTAS) short form [12]. The LCPLTAS consists of seven main domains: (1) basic monetary skills, (2) cash transactions, (3) bank statement management, (4) bill payment, (5) financial conceptual knowledge, (6) financial decision making, and (7) knowledge of personal assets [12]. The depressive symptomatology was measured by the *Geriatric Depression Scale* (GDS-15) [46], and the functionality evaluation was provided by the *Functional Rating Scale for Symptoms of Dementia* (FRSSD), which measures activities of daily living (ADLs) [47]. The above three instruments, along with the MMSE scale, are commonly used by psychiatrists, neuropsychologists, and neurologists in Greece [12,48,49,50,51]. The reported scores were available for all participants in this sample, since they were important part of assessment protocols in medical settings. Note that in the data set there were no missing values.

### 2.3. Method

Cusp analysis was carried out via a modeling procedure based on the probability function, *pdf*, of the dependent measure (Equation (2)):

(2) $$pdf(y)=\xi \exp\left[-\frac{1}{4}y^{4}+\frac{1}{2}by^{2}+ay\right]$$

As the optimization method, the maximum likelihood [52] was used, while the *pdf* was obtained from empirical data. The analysis was performed in R via the cusp package [53]. The cuspfit algorithm utilizes numerical procedures for parameter estimates by minimizing a negative loglikelihood function, on which the model-fit evaluation is based, along with the indices: AIC (Akaike’s information criteria), corrected AIC, and BIC (Bayesian information criteria) and the statistically significant coefficients of the model. Moreover, a comparison of the cusp with the linear and logistic alternative model is provided [53]. The literature offers other modeling procedures as well, such as the GEMCAT methodology [54] and a method implementing Equation (2) and least squares as the optimization method [55]. The details of these methods could be found in a lucid review elsewhere [56].

In the cusp analysis, the financial capacity was the dependent measure (LCPLTAS), known as the state variable, while the depressive symptomatology measured by the *Geriatric Depression Scale* (GDS-15) and the *Functional Rating Scale for Symptoms of Dementia* (FRSSD) were the two control variables. Results from a power analysis [57] (power levels of 80%, a medium effect size, two-tailed test with alpha = 0.05, required sample size of 75) showed that the available sample (*N* = 418) is adequate for testing the multivariate effects under study.

Initially, the model was conceived with the FRSSD as the asymmetry factor and the *Geriatric Depression Scale* (GDS-15) as the bifurcation. The conceptual and mathematical model, however, considers that asymmetry and bifurcation factors that represent antagonistic processes can be operationalized by a combination of the proposed controls, and, consequently, linear functions of the FRSSD and GDS scales were tested as contributing factors to both the asymmetry and bifurcation. The alternative cusp catastrophe models utilize rotated axes [58] and are analogous to the conflict cusp model that has been proposed for Piaget’s conservation task [59]. This cusp model implements (FRSSD − GDS) and (FRSSD + GDS) as asymmetry and bifurcation, respectively.

## 3. Results

Table 1 presents the descriptive statistics (means, standard deviations, minimum and maximum values) for the variables under study. For financial capacity, the LCPLTAS and its short version sLCPLTAS were used. 

Table 2 depicts the correlation matrix for the above variables. Both tables include age and measures of the MMSE, which, however, were not used in the present analysis. Note that both the GDS (*r =* −0.220, *p* < 0.001) and FRSSD (*r =* −0.792, *p* < 0.001) are negatively correlated with financial capacity. 

Subsequently, cusp catastrophe analysis was carried out, testing a model with the FRSSD and GDS as control variables (*Cusp 1*) and a model with the linear combination of them (*Cusp 2*). Table 3 and Table 4 show the slopes, standard errors, Z-tests, and model fit statistics for the cusp and the alternative models.

### 3.1. Cusp 1

In Cusp 1 (Table 3), the FRSSD acts as the asymmetry factor (*b* = −1.4557, *p <* 0.001) and the GDS acts as the bifurcation factor (*b =* −0.3490, *p <* 0.001). The chi-square test of the linear vs. cusp model gives *χ*^2^ = 247.0, *df* = 2, and *p* < 0.001, and the model fit statistics in terms of AIC, AICc, and BIC favor the cusp catastrophe model. The values for the cusp model (AIC = 538.190, AICc = 538.392, and BIC = 562.404) are minimum compared to the linear (AIC = 781.203, AICc = 781.300, and BIC = 797.345) and logistic (AIC = 744.210, AICc = 744.351, and BIC = 764.388) models, respectively. The values of pseudo *R*^2^ are close, but this index is not reliable, and it is not interpreted as the usual percentage of variance explained.

**Figure 2:**
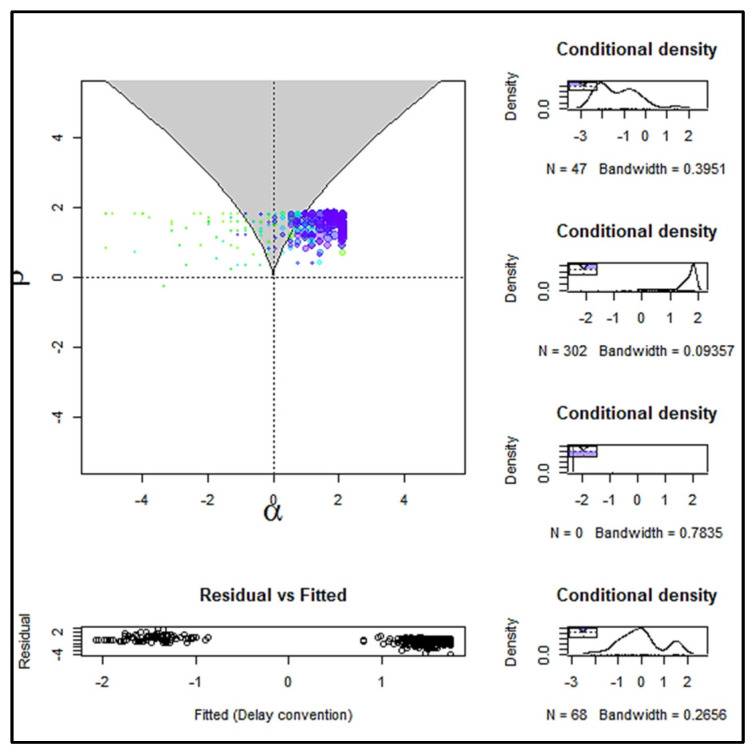
A visual display of the lower part of the cusp response surface of financial capacity using maximum likelihood estimation. FRSSD is the asymmetry factor and depressive symptomatology (GDS-15) is the bifurcation factor.

**Figure 3:**
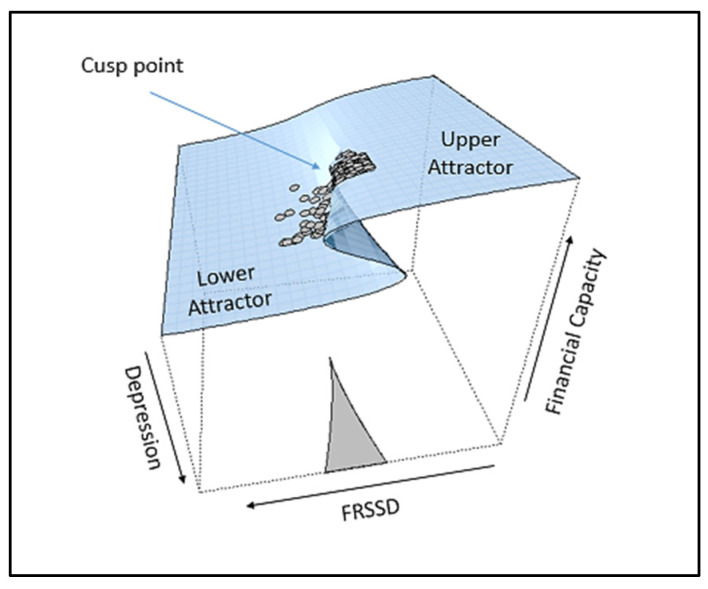
Three-dimensional cusp response surface financial capacity using maximum likelihood estimation. FRSSD is the asymmetry factor and depressive symptomatology (GDS-15) is the bifurcation factor. The gray dots represent observed values from empirical data.

Figure 2 is a visual display of the lower part of the cusp surface, where the shaded region is the bifurcation area. If at least 10% of the observations fall within this area, it is considered as evidence supporting the cusp model [60]. The size of the dots in Figure 2 is a function of the observed bivariate density of the bifurcation factor’s values at that point’s location, and the color is evocative of their position relative to the distance between the two parts of the surface (two attractors), i.e., the observations that are darker in color indicate that they are on or closer to the upper attractor and the observations that are lighter in color are on or closer to the lower attractor. Finally, Figure 3, which depicts the three-dimensional cusp surface as a function of the two control variables, provides an additional visual support for the cusp model, showing that the observations are located at the upper and the lower surface, but not within the area of inaccessibility.

### 3.2. Cusp 2

This cusp catastrophe model utilizes rotated axes [58], analogous to the conflict cusp model [59] using the axes *m* and *n* depicted in Figure 1. It implements a combination of the initially proposed controls, specifically their difference (FRSSD − GDS) and their sum (FRSSD + GDS), as asymmetry and bifurcation factors, respectively.

In Cusp 2 (Table 4), (FRSSD − GDS) acts as the asymmetry factor (*b* = 1.3450, *p* < 0.001) and (FRSSD + GDS) as the bifurcation factor (*b =* 1.2804, *p* < 0.001). The chi-square test of the linear vs. cusp model gives *χ*^2^ = 147.6, *df* = 2, and *p <* 0.001, and the model fit statistics in terms of AIC, AICc, and BIC favor the cusp catastrophe model. The values for the cusp model (AIC = 271.213, AICc = 271.801, and BIC = 289.277) are minimum compared to the linear (AIC = 414.767, AICc = 415.043, and BIC = 426.809) and logistic (AIC = 395.039, AICc = 395.456, and BIC = 410.093) alternatives. The values of pseudo *R*^2^ are 0.63, 0.39, and 0.47 for the cusp, linear, and logistic models, respectively; however, they are not counted in the assessment criteria. 

**Figure 4:**
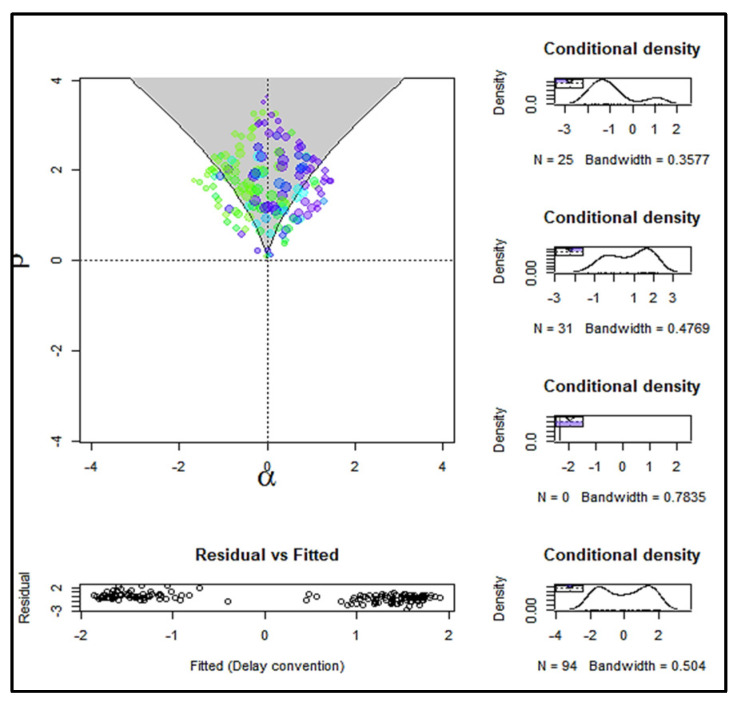
A visual display of the lower part of the cusp response surface of financial capacity using maximum likelihood estimation. (FRSSD − GDS) and (FRSSD + GDS) act as asymmetry and bifurcation factors, respectively.

**Figure 5:**
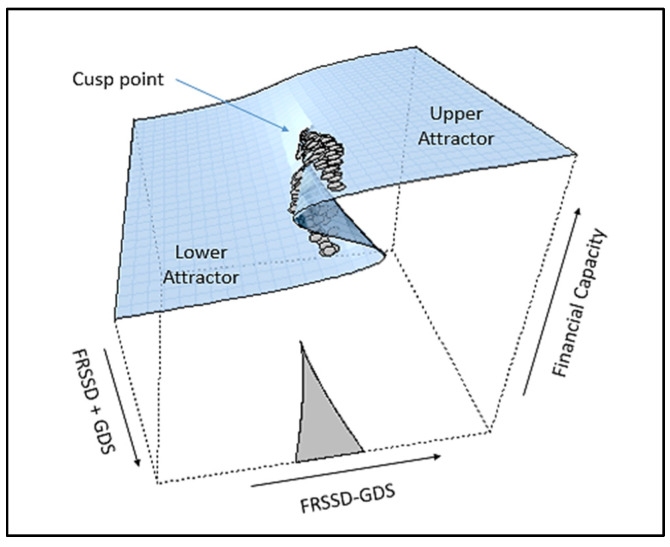
Three-dimensional cusp response surface financial capacity using maximum likelihood estimation. (FRSSD − GDS) and (FRSSD + GDS) act as asymmetry and bifurcation factors, respectively. The gray dots represent observed values from empirical data.

Figure 4, as the visual display of the lower part of the cusp surface, shows that most points are located within the shaded region, the bifurcation area, and in both attractors, the upper and the lower. Figure 5, which depicts the three-dimensional cusp surface as a function of the two control variables, clearly reveals the bifurcation structure with the two diverging slops that are joined at the cusp point and are spreading in each attractor area, while no observations are located in the area of inaccessibility.

### 3.3. Model Interpretation

For *Cusp 1*, which implements the FRSSD and GDS as control variables (Figure 3), the interpretation of the model suggests that, at low values of depression, changes in the state variable (the financial capacity) occur in a smooth and linear manner. In this region, the linear relationship between the state variable and the asymmetry factor, the FRSSD, holds. At higher values of depression, that is, as approaching the forward-facing part of the surface, where surface folds and two behavioral attractors appear, the changes occur merely as transitions between the two attractors. In this region, people with the same control-factor values can be found at the lower and/or at higher attractor regions. This introduces unpredictability in the system and implies that changes in behavior occur as sudden jumps between two modes. 

*Cusp 2* has an analogous interpretation. Note that both the FRSSD and GDS are negatively associated with the financial capacity. When their difference is large, the negative effect is smaller, and as it increases the outcome increases as well. These changes are expected to be smooth and linear compared to the effect of their sum (FRSSD + GDS). When the net moderating effect of the combined high FRSSD and high GDS becomes unexpectedly increased, in that scale, a threshold value is likely to exist, beyond which abrupt changes occur, inducing bifurcation effects. The present analysis supports the above-described roles by providing empirical evidence and establishes catastrophe phenomena in this type of neuropsychological data. It is imperative to repeat that bifurcations and hysteresis effects are complex phenomena, due to the dynamics of the systems and due to self-organization mechanisms. 

## 4. Discussion

The present catastrophe theory model, to our knowledge, is the first application reported in the domain of neuropsychology, and it has twofold implications. The first is epistemological and regards the underlying theory, while the second concerns measurement issues, classifications, and decision-making. The identification of bifurcation effects challenges the epistemological assumptions that adhere to the linear and mechanistic views of a neuropsychology system. Given that bifurcations only characterize CASs [38], their detection indicates that the underlying system is ontologically a complex adaptive system, and it should be investigated as such, i.e., the linear modeling is inadequate and epistemologically incompatible to describe and interpret the system’s behavior [18]. The cusp catastrophe model designated discontinuous changes in a neurocognitive system under gradual changes in the two independent variables, the control factors, namely the asymmetry and the bifurcation. The discontinuous changes occur as transitions between two attractors, which for a neurocognitive system might represent qualitatively distinct modes of behavior, such as a high or low/suboptimal level of performance. 

Note that the present cusp analysis was applied to cross-sectional data, but the interpretation of the model also needs to be extended for the dynamical path of the single case. The individual’s mind involved in a cognitive task, ontologically acting as a CAS, follows a trajectory driven by self-organization mechanisms and the outcome emerges via a dynamical iterative process [21,61,62]. Bifurcations potently occur in those systems and the interpretation of the present model suggests that, in the course of such a dynamical process, even small random fluctuations in the parameters can induce sudden, unexpected transitions from a state of high performance to a state of failure. 

It is pertinent to mention here that bifurcations can be observed and captured analogously when a single case (*N* = 1) is analyzed. Catastrophe phenomena might be relevant and worth examining when dynamical processes are investigated via time series, where nonlinear methods and tools should be employed. Complexity theory offers a theoretical framework and a rich array of methodological tools to support research designs and data analysis. Even though the present investigation used a large sample and cross-sectional data to infer nonlinearity, the effective methodological approach to study CASs is time series analysis [63,64]. This framework has been fruitfully applied in many process approaches [65,66,67,68], where bifurcation phenomena are theoretically anticipated and are worth examining. In those cases, catastrophes of this kind represent changes: cognitive, attitudinal, shifts to coherence, or therapeutic changes. Relevant also is the notion of *ergodicity* in a time series of the analysis [69]. Sudden shifts, transitions, and discontinuities denote a *non-ergodic process,* and the present cusp catastrophe structure supports this idea in neuropsychological data.

What has been learned for the AD and aMCI research, is that depression is not merely a linear moderator of mental operators, but it also reacts with other neurocognitive resources and prompts nonlinear effects. To further stimulate a discussion that will bridge the mathematical/methodological domain with the theoretical premises of neuropsychology, it would be pertinent to think and reflect on the role of other coexisting conditions (e.g., diabetes, heart disease) or other factors of biological and/or psychological origin. The common methodological thought suggests that, in addition to the present choices, additional variables could be included in the cusp model specification and tested with empirical data. The effect of additional candidates is an open issue for further research. However, there are some more interesting aspects to reflect on. 

A pertinent epistemological remark is that a bifurcation effect should be perceived as a process, where the relevant variables being tested, as factors contributing to the underlying mechanism. Under the CAS perspective, this self-organization mechanism concerns the evolution of an interaction system that possibly includes both biological and psychological factors within a *mutual causality* connective state. The representation of such a system is explicitly the ontology of networks, which is in line with complexity theory assumptions. The proper methodology for this is the *network analysis*, where the latest advances offer a better way to approach and understand those systems [70,71,72] compared to the traditional methods. In addition, the network ontology explains the possibility of linear and nonlinear changes, and thus catastrophe theory is on the scene. 

Another interesting remark that is explicated by catastrophe theory is that in the vicinity singularities, e.g., the bifurcations and discontinuities, the behavior could be described merely by a small number of variables. In the present context, depression is one of them. The levels of depression (GDS), even though might be affected by the dynamic interplay of other factors (biological, medical, and/or psychological), contribute to operationalization of the ensuing bifurcation mechanism, in conjunction with the functional symptoms of dementia (FRSSD).

The existing cusp structure in the data and the operating critical points beyond which nonlinear effects occur, directly concern the measurement issues and the relevant theory. The determination of such thresholds is an open issue and of paramount importance in the actual utilization of the *Legal Capacity for Property Law Transactions Assessment Scale* (LCPLTAS) and financial decision-making. In addition, given that the actual bifurcation process is induced by a composite variable, the determination of the critical point is a challenge. The issue is important because it concerns the measurement processes, diagnosis, and further prevention and treatment. 

There are of course limitations in this study, originating from its exploratory character, and since it is the first report with neuropsychological data, the findings should be replicated and extended with other data sets. Cusp analysis could also be tried in other neurocognitive assessments, such as for Parkinson’s disease, and in other neurocognitive assessment tools, such as the MMSE or HoNOS and GAF, to extend the model to different socio-medical inquiries. The present report sets a framework for the application of catastrophe theory with neurocognitive resources in AD research and opens new avenues for investigations. 

Last, but not least, the message that the present findings convey is mainly epistemological and concerns the adoption of the meta-theoretical framework of CASs, the paradigm shift that is gaining ground in interdisciplinary research.

## Figures and Tables

**Table 1:** Descriptive statistics.

	Mean	Std. Deviation	Minimum	Maximum
LCPLTAS	160.605	63.550	0.000	212.000
sLCPLTAS	108.708	43.850	0.000	144.000
MMSE	24.892	6.538	0.000	30.000
GDS	2.725	3.561	0.000	21.000
FRSSD	4.641	6.454	0.000	32.000
Age	72.555	8.061	45.000	98.000

**Table 2:** Pearson’s correlations.

Variable	LCPLTAS	sLCPLTAS	FRSSD	GDS	MMSE	Age
1. LCPLTAS	1					
2. sLCPLTAS	0.998 ***	1				
3. FRSSD	−0.792 ***	−0.789 ***	1			
4. GDS	−0.220 ***	−0.223 ***	0.281 ***	1		
5. MMSE	0.944 ***	0.942 ***	−0.824 ***	−0.201 ***	1	
6. Age	−0.288 ***	−0.289 ***	0.246 ***	−0.018	−0.291 ***	1

* *p* < 0.05, ** *p* < 0.01, *** *p* < 0.001.

**Table 3:** The cusp model estimated by maximum likelihood method: slopes, standard errors, Z-tests, and model fit statistics for cusp and the alternative models. Financial capacity as a function of FRSSD (asymmetry) and Geriatric Depression Scale (bifurcation variable).

Model			b	seb	Z-Value
Cusp 1					
a(Intercept)			1.0628	0.1248	8.52 ***
a[FRSSD]	Functional Rating Scale for Symptoms of Dementia		−1.4557	0.1468	−9.91 ***
b(Intercept)			−1.5417	0.2165	−7.12 **
b[GDS]	Depression Scale		−0.3493	0.0912	−3.83 ***
w(Intercept)			0.8830	0.0355	24.87 ***
w(FC)	Financial Capacity		1.2059	0.02921	41.28 ***
Models’ fit statistics (chi-square test of linear vs. cusp model: *χ*^2^ = 247.0, *df =* 2, *p* < 0.001)					
**Model**	**Pseudo*-R*^2^**	**Npar**	**AIC**	**AICc**	**BIC**
Linear model	0.61	4	781.203	781.300	797.345
Logistic model	0.61	5	744.210	744.351	764.388
Cusp model	0.63	6	538.190	538.392	562.403
Note: *** *p* < 0.001, ** *p* < 0.01, * *p* < 0.05, ^†^ *p* < 0.05 (one-tailed); ns = non-significant.					

**Table 4:** The cusp model estimated by maximum likelihood method: slopes, standard errors, Z-tests, and model fit statistics for cusp and the alternative models. Financial capacity as a function of (FRSSD − GDS) as asymmetry and (FRSSD + GDS) as bifurcation variable.

Model			b	seb	Z-Value
Cusp 2					
a(Intercept)			−0.1606	0.1096	−1.46 ns
a[FRSSD − GDS]	Functional Rating Scale for Symptoms of Dementia		1.3450	0.2181	6.19 ***
b(Intercept)			0.98163	0.3388	2.90 **
b[GDS + FRSSD]	Depression Scale		1.2804	0.1886	6.79 ***
w(Intercept)			0.02605	0.0528	0.50 ns
w(FC)	Financial Capacity		1.02722	0.0472	21.75 ***
Models’ fit statistics (chi-square test of linear vs. cusp model: *χ*^2^ = 147.6, *df* = 2, *p* < 0.001)					
**Model**	**Pseudo*-R*^2^**	**Npar**	**AIC**	**AICc**	**BIC**
Linear model	0.39	4	414.767	415.043	426.809
Logistic model	0.47	5	395.039	395.456	410.093
Cusp model	0.63	6	271.213	271.801	289.277
Note: *** *p* < 0.001, ** *p* < 0.01, * *p* < 0.05, ^†^ *p* < 0.05 (one-tailed); ns = non-significant.					

## Data Availability

The data presented in this study are available upon request from the corresponding author.
